# Polymer Physics Predicts Conformational Diversity of Individual Chromatin Molecules

**DOI:** 10.3390/molecules31213870

**Published:** 2026-10-27

**Authors:** Florinda Di Pierno, Ciro Di Carluccio, Matteo Olimpo, Gabriele Marotta, Mario Nicodemi, Mattia Conte

**Affiliations:** 1 Dipartimento di Ingegneria Chimica dei Materiali e della Produzione Industriale (DICMaPi), Università di Napoli Federico II, Piazzale Vincenzo Tecchio 80, 80125 Naples, Italy; florinda.dipierno@na.infn.it (F.D.P.); ciro.dicarluccio@na.infn.it (C.D.C.); 2 INFN Napoli, Complesso Universitario di Monte Sant’Angelo, 80126 Naples, Italy; matteo.olimpo@unina.it (M.O.); gabriele.marotta@unina.it (G.M.); 3 Dipartimento di Fisica, Università di Napoli Federico II, Complesso Universitario di Monte Sant’Angelo, 80126 Naples, Italy

**Keywords:** chromatin architecture, conformational diversity, conformational heterogeneity, single cell, polymer physics, computational modeling

## Abstract

Recent advances in single-cell and super-resolution imaging have revealed that chromatin adopts highly heterogeneous and dynamic three-dimensional conformations across individual cells. However, the extent of this variability, its underlying mechanisms, and its functional consequences remain difficult to quantify experimentally. Here, we review recent progress in combining polymer physics, molecular dynamics simulations, and data-driven approaches to reconstruct and predict ensembles of three-dimensional chromatin conformations at the single-molecule level. We discuss how polymer phase-separation and loop-extrusion models can reproduce both population-averaged contact data and independent single-cell imaging measurements. These approaches help disentangle different sources of conformational diversity, including the intrinsic thermodynamic degeneracy of polymer folding and cell-to-cell heterogeneity. We further discuss how perturbation of key architectural factors, particularly cohesin, affects chromatin conformation and single-cell structural variability. Overall, polymer-physics models provide a quantitative framework to connect molecular mechanisms underlying chromatin folding with the heterogeneous conformational ensembles observed experimentally in individual molecules.

## 1. Introduction

The three-dimensional (3D) organization of chromatin plays a central role in controlling genome function, such as gene regulation [1,2,3,4,5,6]. Within mammalian cell nuclei, chromosomes fold into a complex, hierarchical architecture that encompasses multiple genomic scales, from local loops [7,8,9] and topologically associating domains (TADs) [10,11,12] to A/B compartments [7,13] and megabase-wide chromosome territories [14]. Population-based approaches, including chromosome conformation capture [15], Hi-C [13,16,17], and ligation-free methods such as GAM [18,19] and SPRITE [20,21], have been instrumental in uncovering these folding principles by providing genome-wide maps of chromatin contacts. However, because these measurements are typically averaged over large cell populations, they do not directly capture the conformations adopted by individual chromatin molecules [4,22,23].

Complementing these population-based approaches, advances in single-cell and super-resolution multiplexed FISH imaging [16,22,24,25,26,27,28,29,30,31] have enabled chromatin conformations to be examined directly in individual nuclei, revealing extensive structural heterogeneity and dynamics across cells. These experiments have shown, for instance, that TAD-like domains and globular structures can be detected at the single-molecule level, although their genomic positions, shapes and interaction patterns can vary from one cell to another [22,28,32,33,34]. Consequently, the domains observed in population-averaged contact maps do not correspond to a unique chromosome structure shared by all cells but rather emerge from heterogeneous ensembles of individual conformations. This structural variability may also have functional consequences, as differences in chromatin folding can modulate the spatial proximity between genes and regulatory elements and thereby contribute to cell-to-cell variation in gene regulation [30,33,35,36]. Consistently, genomic mutations that disrupt TAD boundaries, such as structural variants altering chromosome folding, have been linked to changes in gene misexpression and disease [37,38,39,40,41,42,43,44,45,46]. Therefore, understanding the physical mechanisms that generate this conformational diversity, and how such structural variability can coexist with specific and functionally relevant genomic interactions, has become a central challenge in chromosome biology.

To address this question, a variety of quantitative models grounded in polymer physics have been developed to identify the molecular and physical mechanisms that shape chromosome conformations [8,25,47,48,49,50,51,52,53,54,55,56,57,58,59,60,61,62,63,64,65,66,67,68,69,70,71,72]. Two broad classes of models have received particular attention because their minimal molecular ingredients have been comparatively well characterized. Loop-extrusion (LE) models [8,47,48,49,67,68] describe chromatin folding as an active, out-of-equilibrium process in which molecular complexes, such as cohesin, translocate along the chromatin fiber, progressively enlarging loops until extrusion is halted, for example, by convergently oriented CTCF sites. A second class of models relies on preferential interactions among chromatin regions, which drive their spatial segregation and the formation of compact domains through polymer phase separation (PS) [50,51,52,53,55,56,57,58,59,60,61,62,73,74]. In these models, the polymer is partitioned into distinct monomer or chromatin types based on experimentally determined biological (e.g., epigenetic) information, and type-specific interactions drive their spatial segregation.

The Strings and Binders (SBS) model, whose predictions have been recently tested against a broad range of independent experimental datasets, including population-averaged Hi-C and GAM maps, protein-mediated contacts measured by HiChIP and ChIA-PET, and chromatin structures resolved by single-cell FISH microscopy [16,22,26,28,32,75,76], also belongs to the interaction-driven PS class [60,61,62]. In the SBS model, chromatin is represented as a self-avoiding polymer carrying different classes of binding sites that interact through cognate molecular binders. Differently from other approaches, the genomic arrangement of these binding sites can be inferred directly from experimental contact maps, without requiring epigenetic annotations as prior information [61,62]. Despite differences in their formulation and molecular interpretation, PS-based models share, nevertheless, the central principle that chromosome organization can emerge through preferential interactions among specific chromatin states or binding domains.

Beyond these specific folding mechanisms, chromosome conformations are also shaped by the physical environment of the cell nucleus. Chromosomes are densely packed within a confined nuclear volume, where confinement and topological constraints between chromatin fibers contribute to the formation of compact, territorial structures [77,78,79,80,81,82]. Macromolecular crowding can further favor compaction and stabilize folded states through entropic depletion effects [83], while interactions with the nuclear lamina tether large chromatin domains to the nuclear periphery in a cell-to-cell variable manner [84,85,86,87], and heterochromatin interactions drive the large-scale compartmentalization of the nucleus [88]. These factors can reshape the accessible conformational landscape of chromatin and modulate its cell-to-cell variability.

Within this broader physical context, here we examine the intrinsic folding mechanisms of individual genomic loci, a scale at which minimal polymer-physics models already capture key features of single-cell structures. We discuss how these models can be used quantitatively to interpret and predict the broad conformational diversity of individual chromatin molecules, as revealed by recent microscopy studies. To this end, we focus on loop extrusion and the SBS model as representative active and PS-based mechanisms, respectively. We discuss the physical mechanisms through which they generate structural variability across individual conformations and examine how faithfully they reproduce population-averaged and single-cell measurements. As an illustrative case, we consider a 2.5 Mb locus (chr21:34.6–37.1 Mb, hg38) in human HCT116 cells, hereafter referred to as the HCT116 locus for brevity, for which both population-averaged Hi-C [9] and multiplexed single-cell imaging data [22] are available. We show that the models capture not only average contact and spatial-distance maps, but also non-trivial properties of individual chromatin structures, including the distributions of TAD-like boundaries in single cells, spatial segregation, and cell-to-cell conformational variability, as validated by single-cell microscopy data [22,61,62]. We then examine how perturbations of key architectural factors, particularly cohesin depletion, can alter chromatin folding and its structural heterogeneity, illustrating how polymer models can connect specific molecular mechanisms of folding to the diversity of 3D conformations observed experimentally.

Overall, these studies demonstrate that polymer-physics models can move beyond the reconstruction of average chromosome architecture to predict the heterogeneous ensembles of conformations adopted by individual chromatin molecules. By linking molecular mechanisms, 3D structure, and cell-to-cell variability within a unified quantitative framework, these approaches provide a basis for predicting how chromatin organization is affected by disease-associated mutations, altered regulatory states, or targeted perturbations, even when the resulting single-cell structures are not yet directly accessible experimentally.

## 2. Physical Mechanisms of Chromatin Folding

### 2.1. Loop-Extrusion Model

Loop extrusion provides a physical mechanism for the formation of chromatin loops and topologically associating domains based on the active translocation of molecular complexes along the chromatin [8,47,48,49,67,68]. According to the model, loop-extrusion complexes bind to chromatin and progressively enlarge a loop by moving along the polymer until extrusion is interrupted by dissociation, collision with another extruder, or encounter with specific anchor-sites (Methods, Section 7). In mammalian cells, cohesin has been identified as a major component of the loop-extruding machinery, while CTCF-binding sites with convergent orientation can promote the formation of specific loops and contribute to the positioning of TAD boundaries (Figure 1, left) [8,47,49,67,89,90,91,92,93,94,95]. Because translocation requires an energy supply, loop extrusion is intrinsically an active and out-of-equilibrium folding mechanism [49,96,97].

Polymer implementations of loop extrusion reproduce this process by introducing dynamic bonds or springs that connect progressively more distant sites along the chain as extrusion proceeds [8,62,67]. The stochastic binding and dissociation of extruders naturally generate an ensemble of polymer conformations rather than a single deterministic structure. In the simplest implementations [8,62,67], anchor-site positions and their orientations are fixed and shared among all molecules. More refined implementations can introduce variability in anchor-site occupancy across individual conformations, assigning each site a probability of being present in each molecule [57,62]. Such extensions provide a way to account for cell-to-cell differences in the molecular landscape experienced by chromatin and can improve the ability of LE-based models to reproduce experimental contact and distance patterns at both the population and single-molecule levels.

Thus, although the physical principle of the model is the active extrusion of loops, the resulting chromatin architecture depends on the combined dynamics of extruders, polymer fluctuations, and the genomic distribution and accessibility of their anchor-sites. This model provides a direct mechanistic connection between cohesin, CTCF-binding sites, transcriptional regulation and the emergence of chromatin loops and domain boundaries, while structural variability arises from the stochastic nature of the extrusion process and from molecular heterogeneity across individual cells [49,62,98].

### 2.2. Phase-Separation-Based Models

A different class of polymer models of chromatin describes folding as the consequence of preferential interactions between specific DNA regions rather than relying on an active motor that brings distal loci together. This class of models encodes different biochemical or epigenetic identities along the polymer and assigns different interaction affinities to the corresponding chromatin types [51,54,55,60,61,62,74,77,99]. Attractive interactions between compatible regions, either direct or mediated by diffusing protein-like bridging binders [100,101,102,103], can then drive their preferential interaction and spatial segregation, producing compact domains and compartmentalized chromosome conformations through the statistical mechanics of self-interacting polymers [50,51,53,54,55,56,57,58,60,61,64,65,73,104,105,106,107,108]. Despite substantial differences in their microscopic implementation, these approaches share the general principle that heterogeneous 1D sequence of chromatin binding sites can be translated into a non-random 3D architecture through type-dependent interactions [109,110,111,112].

Several PS-based polymer models have been proposed to describe chromatin organization. For example, block-copolymer models [55], the Chromosome Copolymer Model (CCM) [58,59], MiChroM [51,52,113], and related approaches [50] represent chromatin as a heteropolymer composed of distinct monomer types, often defined from input epigenetic information, whose preferential interactions drive domain formation, microphase segregation, and heterogeneous 3D conformations at equilibrium. These interactions may be implemented either as direct attractions between chromatin segments or through multivalent molecules that bridge distant cognate sites [51,54,55,56,57,58,60,61,62,74]. In the latter case, bridging can increase the local concentrations of both binding sites and binders, promoting further interactions through a positive-feedback mechanism known as bridging-induced phase separation [54,56,114]. Despite their different microscopic implementations, these models belong to the same broad physical class of self-interacting heteropolymers and can therefore exhibit analogous coil-to-globule transitions and spatial segregation of interacting domains [55,62,74,115].

The Strings and Binders (SBS) model provides a particularly general and tractable realization of this interaction-driven scenario. Chromatin is represented as a self-avoiding-walk (SAW) polymer carrying different classes of binding sites, while diffusing cognate molecules bridge sites of the corresponding type (Figure 1, right) [60,61,62]. Its conformational folding states, corresponding to the system thermodynamic phases, are governed by the competition between polymer entropy and binder-mediated attractions. At low binder concentrations or affinities, entropy dominates and the polymer remains in an open (“coil”) state belonging to the SAW universality class [104,116]. Above a characteristic interaction threshold, binder-mediated attractions overcome the entropic cost of compaction, driving a switch to a globular state in which distinct classes of binding sites assemble into spatially segregated domains [115,117,118]. A key feature of the SBS model is that the genomic positions and identities of its binding sites need not be prescribed from prior epigenetic annotations. Instead, they can be inferred directly from experimental contact maps using numerical optimization methods [61,119,120] (Methods, Section 7). The inferred binding domains can then be correlated with chromatin-associated factors and epigenetic features, including CTCF/Cohesin and active or repressive histone marks, providing testable molecular predictions on their combinatorial mode of action [61,62,74].

## 3. Polymer Folding at the Population-Averaged Level

Both LE and PS-based models have been shown to recapitulate key features of population-averaged chromatin architecture, including loops and TAD-like domains, despite relying on substantially different physical mechanisms [8,47,49,50,51,54,55,60,61,62,67]. A direct comparison of these mechanisms has been performed for the 2.5 Mb HCT116 locus (chr21:34.6–37.1 Mb), for which multiplexed FISH measurements provide pairwise spatial distances between genomic loci in individual cells [22,62]. Taking the median distance for each pair of loci across cells produces a population-level distance matrix characterized by distinct domain-like structures (Figure 2a, top panel). Polymer models of the same region were constructed using either LE or SBS interactions, with the genomic distributions of LE anchor-sites and SBS binding domains defining the locus-specific folding constraints (Figure 2a, middle and bottom) [62].

The ensembles generated by the two models were then used to calculate the corresponding median-distance matrices (Figure 2b, top). Remarkably, both mechanisms reproduce the main domain-scale patterns observed experimentally. To quantify this agreement, we use the genomic-distance-corrected Pearson correlation, *r*′, i.e., the Pearson correlation between two matrices computed after subtracting from each entry the average value of the diagonal it belongs to, which removes the trivial dependence of distances on genomic separation (Methods, Section 7). The *r*′ correlations between the experimental matrix and those predicted by the LE and SBS models are *r*′ = 0.87 and *r*′ = 0.84, respectively, substantially higher than the near-zero correlation expected for a randomly folded polymer [62]. Thus, although LE organizes chromatin through dynamically extruded loops, whereas the SBS model relies on preferential interactions among binding domains, both mechanisms quantitatively reproduce its population-level spatial organization.

This agreement extends to the genomic distribution of TAD-like boundaries [22]. The boundary probability measures how frequently a domain boundary occurs at each genomic position across individual conformations, thereby connecting local single-molecule organization to the contact domains emerging in population-level maps [22]. Both models reproduce the non-uniform experimental boundary profile, with Pearson correlations of r = 0.50 for LE and r = 0.79 for SBS (Figure 2b, bottom) [61,62], indicating that both mechanisms capture bulk chromatin structure beyond the overall distance pattern.

The stronger correlation obtained with the SBS model may reflect the broader information encoded by its inferred binding domains. In the LE model, preferential boundary positions arise from CTCF/cohesin-defined anchors, whereas SBS domains effectively represent a broader set of interaction determinants captured by the population-averaged contact matrix, without assigning them *a priori* to specific molecular factors. Importantly, at the same HCT116 locus, microscopy data have shown that TAD-like boundaries occur throughout the region and are only preferentially enriched at a subset of CTCF/cohesin sites [22]. Moreover, cohesin depletion removes preferential boundary positioning and population-averaged TADs but not TAD-like domains in individual cells [22], hinting that additional factors beyond CTCF/cohesin contribute to their formation. Consistently, CTCF/cohesin-independent folding associated with transcription, BRD4, RNA polymerase II, and condensate-mediated interactions has been extensively reported [121,122,123,124]. Fine-scale regulatory interactions also exhibit variable dependence on cohesin and CTCF [125], while enhancer-promoter contacts can bypass CTCF-mediated boundaries [126]. Accordingly, the inferred SBS domains have been shown to correlate with different combinations of CTCF/cohesin, transcriptional regulators, and active or repressive chromatin marks [61,62,119], allowing the model to integrate the effective contributions of multiple chromatin-associated factors and thus potentially explaining its stronger agreement with the experimental boundary profile.

Together, these results show that both LE and SBS models can quantitatively explain the average folding of chromatin, despite their distinct underlying molecular mechanisms. The next critical test is therefore whether they also reproduce the distribution and structural heterogeneity of the individual conformations underlying these population-level patterns. This requires moving beyond ensemble summaries to directly compare experimentally measured and model-predicted single-chromatin conformations [49,62,113,127].

## 4. Conformational Diversity at the Single-Molecule Level

Population-level contact and distance maps provide only a partial description of chromosome organization, because similar average patterns can emerge from different distributions of individual conformations. Multiplexed FISH measurements now enable a more stringent test of polymer models, i.e., whether their predicted ensembles reproduce not only average chromatin architecture but also the structural diversity observed across individual cells. In the HCT116 locus considered here, single-molecule distance matrices exhibit pronounced TAD-like patterns whose positions, sizes, and internal organizations vary substantially from cell to cell [22]. Simulations based on both LE and the SBS mechanism generate a similarly broad diversity of individual structures (Figure 3a) [62], each representing an independent steady-state realization of a polymer model whose input is restricted to population-averaged data only.

This structural heterogeneity can be quantified by the pair correlation, i.e., the *r*′ correlation computed between the distance matrices of two individual conformations, whose distribution over all pairs of molecules within an ensemble measures their structural similarity [61,62,127,128] (Methods, Section 7). The experimental distribution is broad, indicating that different molecules retain a degree of common organization while adopting substantially different conformations. Similarly, the SBS distribution closely matches the experimental reference, with an average *r*′ = 0.22, very close to the corresponding experimental value of *r*′ = 0.24 (Mann–Whitney test, *p* = 0.079; Figure 3b) [22,62]. Importantly, pair correlations are not used to infer the model, nor is any parameter fitted to them: they are computed a posteriori from the ensemble of single-molecule conformations, so that their agreement with the experimental data provides an independent validation of the model at the single-molecule level. Conversely, the LE distribution is shifted toward lower correlations (mean *r*′ = 0.11), showing that LE conformations are, on average, less similar to each other and therefore exhibit a higher degree of structural variability [61,62]. This difference may reflect the fact that the LE polymer remains essentially an open, coil-like self-avoiding chain subject to local constraints imposed by CTCF/cohesin-mediated loops. Owing to the stochastic formation and dissolution of these loops, the polymer can explore a broader range of spatial arrangements, whereas preferential interactions among binding domains in the SBS model drive more compact and structurally persistent globular conformations [60,61,62]. Thus, although both mechanisms reproduce population-level folding, single-molecule comparisons more clearly distinguish the conformational ensembles they generate.

Single-cell heterogeneity also extends beyond local distance patterns to the global geometry of chromatin. At the simplest geometric level, individual chromatin conformations can be characterized by approximating their overall shapes as triaxial ellipsoids and measuring the ratios between their principal semi-axes [22,62,78,129]. Experimental HCT116 structures exhibit broad distributions of the *a*/*b* and *b*/*c* axis ratios, rather than a single stereotyped shape. Both LE and SBS models reproduce the main features of these distributions (Figure 3c), indicating that they capture the anisotropy and shape variability of individual chromatin molecules experimentally observed. In all three ensembles, the predominance of larger *a*/*b* than *b*/*c* ratios is consistent with preferentially prolate, rather than spherical, conformations [62].

Comparable agreement is observed for chromatin compaction. Classifying individual molecules according to their relative volumes reveals a heterogeneous population ranging from more compact to more open structures (Figure 3d). Both models reproduce the experimental fractions across the different compaction classes, showing that their conformational ensembles span a range of volumes comparable to that measured by multiplexed FISH [62]. Together, the pair-correlation, shape, and compaction analyses demonstrate that polymer models can account for multiple independent aspects of genome conformational diversity, beyond the population-level architecture of the locus, providing a mechanistic basis for understanding complex biological functions, such as single-molecule gene regulation [130].

This structural diversity is an intrinsic consequence of the statistical nature of polymer folding and does not necessarily require different interaction rules for every molecule. In the SBS model, thermal fluctuations and the thermodynamic degeneracy of the domain-segregated state allow multiple arrangements of interacting domains with comparable free energies to coexist within the same ensemble. In LE models, stochastic extruder binding, translocation, and dissociation generate different loop configurations across individual molecules. Cell-to-cell differences in molecular constraints, such as anchor-site occupancy or binder availability, can further broaden these distributions [57,61,62,131]. Chromatin heterogeneity can therefore emerge from both the intrinsic stochasticity of polymer dynamics and variations in the molecular factors governing chromosome folding.

## 5. Cohesin Depletion Enhances Single-Molecule Conformational Variability

Perturbing chromatin architectural factors provides a direct means of identifying the molecular mechanisms that control chromosome folding and its cell-to-cell variability. Cohesin depletion, in particular, has been shown to profoundly alter population-level domain organization [9,73,132,133,134]. In HCT116 cells carrying an auxin-inducible cohesin-degradation system, the pronounced domain patterns observed in untreated cells are largely lost, and the population-level distance matrix becomes dominated by the genomic-distance-dependent diagonal signal (Figure 4a) [22,61]. Both LE and SBS models reproduce this more homogeneous average organization, consistent with cohesin contributing to chromatin folding through loop extrusion and, potentially, binder-mediated interactions.

The consequences of cohesin depletion are even more interesting at the single-molecule level. Despite the loss of domains from the population-level matrix, individual HCT116 + Auxin conformations remain heterogeneous and can display non-trivial contact patterns (Figure 4a) [22,61]. Their corresponding *r*′ correlation distributions are broad and centered near zero, indicating little structural similarity between different molecules and greater conformational variability than in untreated cells. Both LE and SBS models reproduce this shift toward broad, near-zero pair-correlation distributions (Figure 4b) [61]. Thus, the loss of a reproducible average domain pattern can coexist with substantial structure and heterogeneity in individual chromatin molecules.

Within the LE framework, cohesin depletion removes the motors that generate chromatin loops, shifting the polymer toward a self-avoiding-walk regime dominated by stochastic contacts [8,49,61,62]. In the SBS model, it weakens binder-mediated interactions that stabilize compact domains, a plausible interpretation given that cohesin can also bridge distant DNA segments and promote DNA-cohesin condensation [61,135]. In both cases, transient local structures may persist at variable genomic positions but disappear upon ensemble averaging, producing a largely featureless population-level map.

After cohesin depletion, heterogeneous single-cell domains remain detectable, but their boundaries lose their preferential genomic localization [22]. Although incomplete depletion cannot be excluded, this persistence is also consistent with cohesin-independent interactions: variable single-cell domains occur, for instance, on both active and inactive X chromosomes and are largely preserved after perturbations of major epigenetic components and transcription [136], suggesting a combinatorial contribution of multiple molecular factors.

Together, perturbation experiments and polymer modeling support a picture in which cohesin plays a major but non-exclusive role in chromosome organization. Cohesin helps establish folding constraints that produce population-level TAD patterns and limit conformational variability, whereas stochastic polymer folding and additional chromatin interactions can sustain heterogeneous domain-like structures after its depletion. Single-cell chromatin organization therefore likely emerges from the combined action of multiple folding mechanisms rather than from cohesin activity alone [61,62].

## 6. Conclusions

The studies reviewed here demonstrate that both loop extrusion and phase-separation-based mechanisms can reproduce key features of chromatin organization at population and single-molecule levels. Although based on distinct physical principles, both generate conformational ensembles consistent with bulk contact maps and microscopy data while capturing non-trivial properties of individual chromatin structures. Their sources of variability, though, differ: phase-separation-based models sample multiple thermodynamically accessible polymer states, whereas loop-extrusion models generate heterogeneity through stochastic extruder dynamics and molecule-to-molecule variation in anchor-site occupancy [61,62].

These mechanisms need not be mutually exclusive. Loop extrusion and polymer phase separation can operate simultaneously on the same chromatin molecule, and models combining them can improve the description of multiple experimental observables, including higher-order contacts [62,66]. Chromosome folding is therefore emerging from an interplay of active and thermodynamic processes rather than from a single dominant mechanism.

Perturbation experiments show that cohesin depletion disrupts population-level domain organization and increases single-molecule conformational variability, yet heterogeneous domain-like structures persist in individual chromosomes [9,22,61]. Together with the association of inferred interaction domains with combinations of architectural proteins and epigenetic features, this persistence suggests that cohesin has a major but non-exclusive role and that multiple molecular factors jointly shape the accessible conformational ensemble [61,62,74].

Beyond the LE and SBS models considered in detail here, a growing number of polymer physics approaches has been integrated with single-cell imaging data, encompassing, e.g., multiplexed FISH, chromatin tracing and ORCA [22,26,137,138]. These include, for instance, polymer models describing TADs as nano-compartments in single Drosophila cells [139], cross-linked chromatin models showing that chromosome dynamics near a sol–gel transition controls the timing of distal genomic encounters [140], generalized Rouse models accounting for the cell-to-cell conformational heterogeneity revealed by FISH [141], maximum-entropy approaches reconstructing ensembles of 3D structures directly from imaged pairwise distances [142], and data-driven models of diploid genome organization validated against imaging measurements [143]. Together, these studies place the LE and SBS models within a broader landscape of polymer models of single-cell chromatin organization, all pointing to structural heterogeneity as a defining feature of chromosome folding.

Future extensions of the models discussed here could explicitly incorporate these additional physical factors, as also explored in other modelling approaches [79,80,81,83,86,87,144,145,146,147,148,149], to assess how they modulate the extent and nature of single-molecule structural heterogeneity.

Overall, this highlights that chromatin conformational diversity is not simply experimental noise around an average structure, but an intrinsic property of chromosome organization. Because distinct mechanisms can produce similar population-level patterns, single-cell measurements are essential for discriminating between alternative physical models. Conversely, polymer models provide the mechanistic framework needed to interpret these heterogeneous structures and connect them to their underlying molecular interactions. Integrating population-level and single-cell data can therefore move polymer modeling beyond the reconstruction of average chromatin architecture toward predicting how genetic variants, epigenetic alterations, and targeted perturbations reshape the full conformational landscape of chromatin and, ultimately, its regulatory function.

## 7. Methods

### 7.1. Experimental Data and Studied Locus

The models discussed in this review refer to the 2.5 Mb locus chr21:34.6–37.1 Mb (hg38) in human HCT116 cells for which multiplexed FISH data provide the 3D coordinates of consecutive 30 kb genomic segments in individual cells [22]. To test the models at the single-molecule level, population-averaged information, i.e., bulk Hi-C data [9], is only used as input, whereas single-cell imaging data are used exclusively for their independent validation [61,62].

### 7.2. Polymer Models

In both LE and SBS models, a chromatin region is represented as a self-avoiding chain of beads of diameter σ, where consecutive beads are connected by finitely extensible nonlinear elastic springs and bead overlap is prevented by a short-range repulsive potential [61,62,150].

In the LE model, loop-extruding factors are modelled as additional harmonic springs that bind to the polymer chain and extrude loops by translocating along it [62,67]. Their number is fixed, they cannot pass through each other, and their translocation halts when they encounter another extruder or an anchor site with opposite orientation, unless they stochastically dissociate from the chain. The positions and orientations of the anchor sites are determined by a standard motif-finding analysis of CTCF-binding sites, named FIMO [151], based on the peaks of CTCF ChIP-seq data from ENCODE [62]. To account for cell-to-cell epigenetic heterogeneity, each anchor site is present in a given single molecule with a finite probability, which is set to best reproduce population-averaged contact and distance data of the locus [62]. Notably, in the HCT116 locus, the anchor sites of the model correspond to a subset of CTCF motif sites, which also overlap with other histone and transcription marks, such as H3K27ac, H3K4me1, H3K27me3 and Pol-II [62], hinting that they could be combined with other chromatin factors to determine loop sites, consistent with recent experimental findings [152,153].

In the SBS model, the polymer chain carries specific binding sites of different types, visually represented by different colours, for cognate molecular binders, as well as unspecific binding sites with lower affinity [61]. The binding affinities are taken in the weak biochemical energy range, i.e., from 3.1 to 8.0 k_B_T for specific interactions and from 0 to 2.7 k_B_T for unspecific ones [61]. Binders are diffusing Brownian particles that interact only with binding sites of the same type and can bridge them, driving the folding of the chain through thermodynamic mechanisms of phase transitions [60,61,62,104]. Specifically, upon increasing the binder concentration (or affinity) above a threshold value, which falls in the μmol/L range, the system undergoes a phase transition from a coil, i.e., randomly folded, state to a phase-separated state, in which distinct globules self-assemble along the chain through the interactions of cognate binding sites [60,61,62].

The genomic positions and types of the SBS binding sites are inferred by PRISMR, a polymer-physics-based machine-learning procedure that takes as input only bulk Hi-C data, without prior epigenetic information [61,119,120]. To account for the possibility of multiple binding sites within each 30 kb window, the model accommodates up to r beads per window, and the optimal value, r* = 10, is estimated by the procedure, corresponding to about 3 kb per bead and 830 beads for the HCT116 locus [61,120]. PRISMR is based on a simulated annealing Monte Carlo optimization that minimizes a cost function, defined as the mean squared difference between the input and model-derived contact matrices, with each term rescaled by the average Hi-C intensity at the corresponding genomic distance [61,120]. To avoid overfitting, the cost function also includes a regularization term penalizing the addition of new binding sites, as extensively discussed in previous works [61,119,120]. This procedure returns four distinct binding-site types for the wild-type HCT116 locus and three for the cohesin-depleted (HCT116 + Auxin) locus [61]. The inferred binding domains can then be compared *a posteriori* with available epigenetic data, by computing, e.g., Pearson correlations between each binding-site type and ChIP-seq tracks binned at the same 30 kb resolution, and assessing their significance against a random control of bootstrapped binding domains. In the HCT116 locus, each binding-site type correlates with a specific combination of chromatin factors, including, CTCF/Smc1 (cohesin), H3K27ac, repressive marks such as H3K27me3, and H4K16ac and specific transcription factors [61].

The 3D conformations of the models are generated by Molecular Dynamics simulations via the LAMMPS software (version 30 July 2016) [154,155], starting from independent self-avoiding-walk configurations and running until stationarity is reached (up to 10^8^ MD time steps) [61,62]. For each model, an ensemble of 10^3^ independent equilibrated single-molecule structures is collected; therefore, each conformation is an independent realization of the polymer model at steady state [61,62]. The models are parametrized using population-averaged experimental data, without fitting individual single-cell conformations: the SBS binding domains are inferred from bulk Hi-C data [9], and the LE anchor sites correspond to bulk CTCF/cohesin-binding sites, with parameters set on population-averaged data. Thus, the resulting ensembles of single-molecule conformations are genuine model predictions, which are tested against independent single-cell multiplexed FISH data [22]. Finally, simulated distances are expressed in units of the bead diameter σ, which is converted into physical units by equating the medians of the model and experimental gyration-radius distributions [61,62]. In the HCT116 locus, this yields σ = 41 nm for the LE model and σ = 45 nm for the SBS model [62]. This calibration only sets the length scale of each model, and no parameter is fitted on single-cell data.

### 7.3. Quantitative Comparison of Conformational Ensembles

All model–experiment comparisons are performed at the 30 kb resolution, to match the experimentally reported resolution of the imaging data [22]. The similarity between two distance matrices (e.g., model and experiment), $D_{\mathrm{ij}}^{\left(1\right)}$ and $D_{\mathrm{ij}}^{\left(2\right)}$, is quantified by the genomic-distance-corrected Pearson correlation *r*′, i.e., the Pearson correlation computed after subtracting from each entry the average value of the diagonal it belongs to:

$$r^{\prime }=\frac{\sum_{i<j}\left(D_{\mathrm{ij}}^{\left(1\right)}-{\bar{D}}_{s_{\mathrm{ij}}}^{\left(1\right)}\right)\left(D_{\mathrm{ij}}^{\left(2\right)}-{\bar{D}}_{s_{\mathrm{ij}}}^{\left(2\right)}\right)}{\sqrt{\sum_{i<j}{\left(D_{\mathrm{ij}}^{\left(1\right)}-{\bar{D}}_{s_{\mathrm{ij}}}^{\left(1\right)}\right)}^{2}\sum_{i<j}{\left(D_{\mathrm{ij}}^{\left(2\right)}-{\bar{D}}_{s_{\mathrm{ij}}}^{\left(2\right)}\right)}^{2}}}$$

where ${\bar{D}}_{s_{\mathrm{ij}}}$ is the average value of the matrix along the diagonal corresponding to the genomic separation *s_ij_* = |*i* − *j*|. Computed between median-distance matrices, *r*′ measures population-level similarity (Figure 2b). Computed between the distance matrices of two individual conformations, it defines the pair correlation, whose distribution over all pairs of conformations within an ensemble quantifies single-molecule structural heterogeneity (Figure 3b and Figure 4b) [61,62,128]. Statistical differences between *r*′ distributions were assessed using a two-sided Mann–Whitney U test, with (*p* < 0.05). Exact *p*-values are reported where applicable.

## Figures and Tables

**Figure 1:**
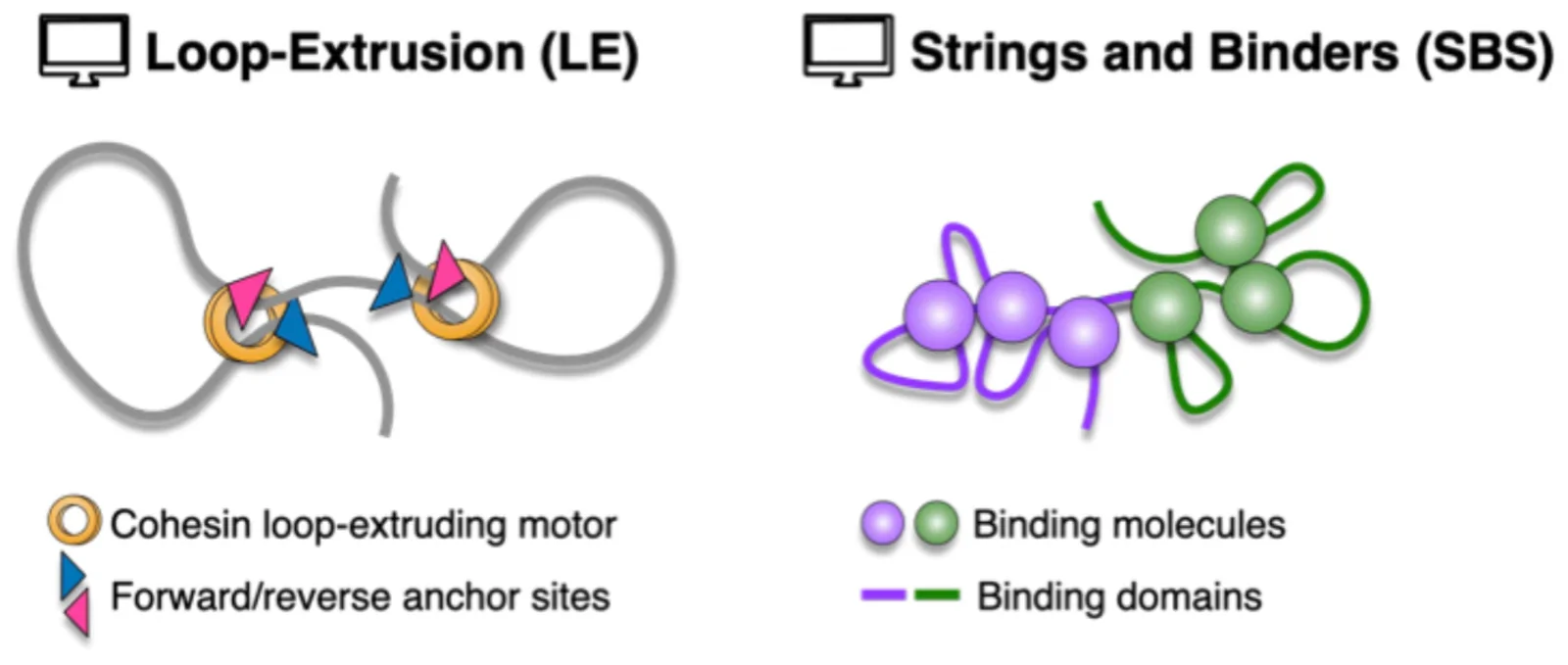
Polymer-physics models of chromatin conformations. Two major physical mechanisms proposed to underlie chromatin folding are illustrated. (**Left**) In the loop-extrusion (LE) model, cohesin complexes act as active motors that extrude chromatin loops until they encounter convergently oriented anchor-sites or other extruders. (**Right**) In the Strings and Binders (SBS) model, chromatin folding is driven by interactions between specific binding domains and cognate binding molecules, promoting the self-assembly of spatially segregated chromatin regions. Adapted from [62].

**Figure 2:**
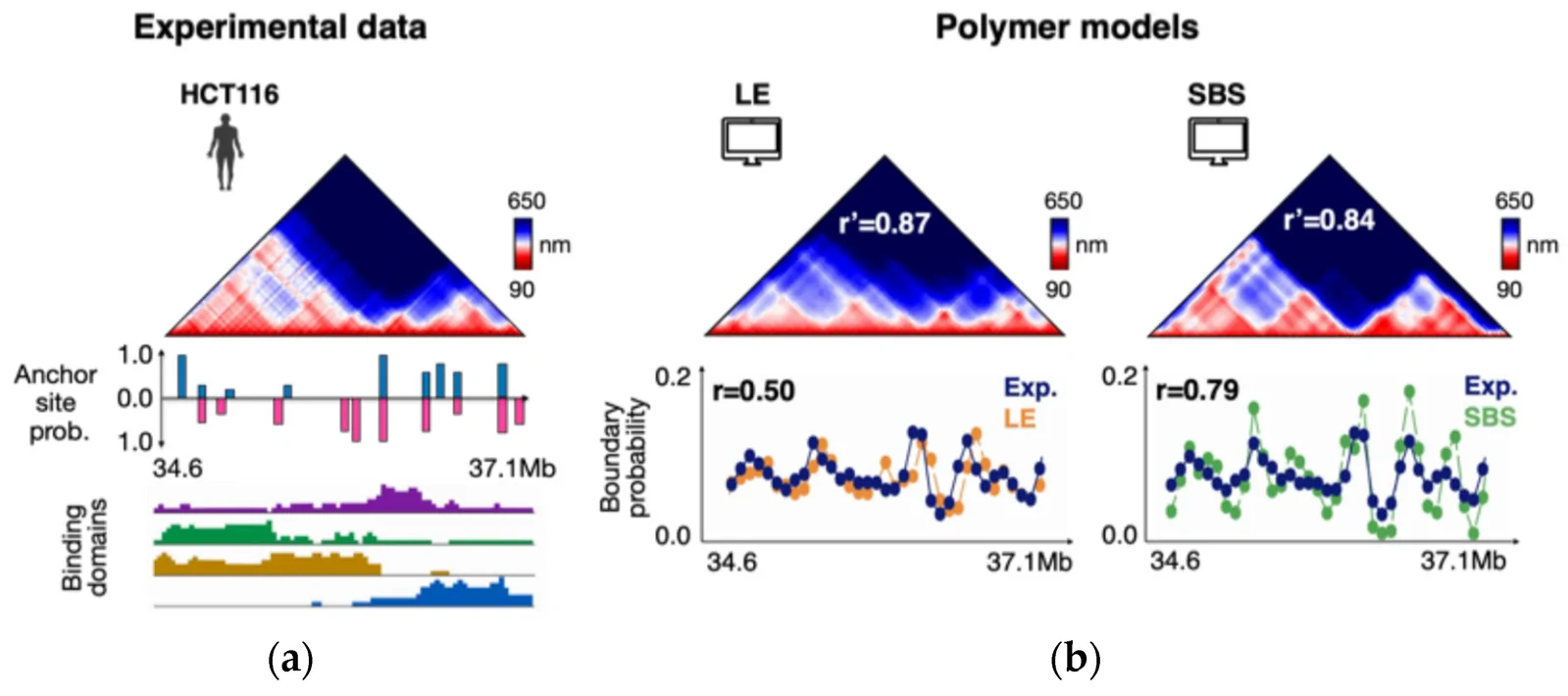
Loop-extrusion and phase-separation-based models reproduce population-averaged data of chromatin structure. (**a**) Experimental median-distance matrix of the 2.5 Mb locus chr21:34.6–37.1 Mb in human HCT116 cells [22], together with the LE anchor-site probabilities and the SBS binding domains. (**b**) Median-distance matrices predicted by LE and SBS polymer models are compared with the experimental data, along with the corresponding boundary-probability profiles. Both models reproduce the main structural features of the locus at the population-averaged level, as quantified by the reported genomic-distance-corrected correlations (*r*′) for the median-distance matrices and Pearson correlations (r) for the boundary-probability profiles. Adapted from [62].

**Figure 3:**
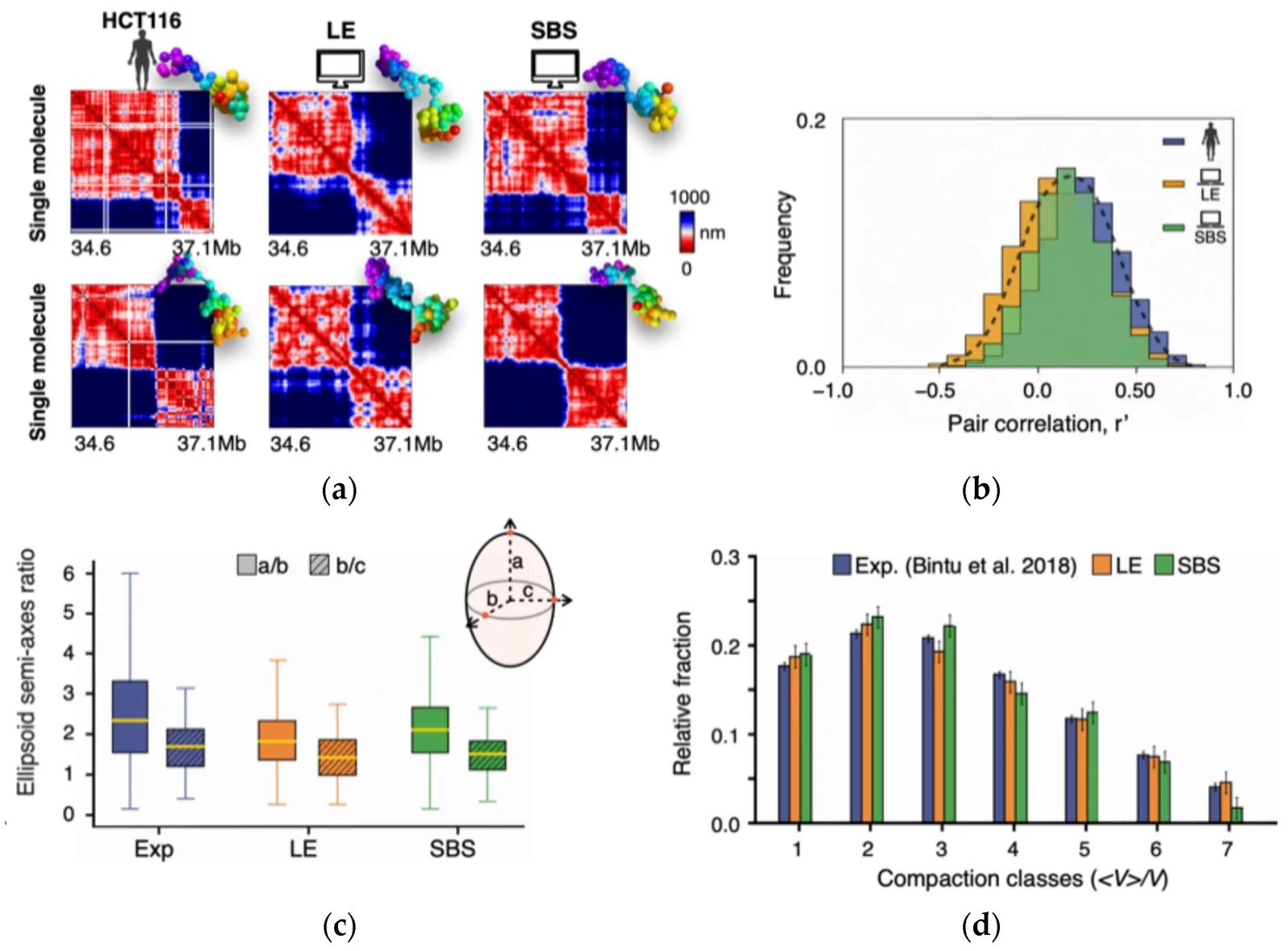
The models predict single-cell chromatin conformations consistent with multiplexed FISH microscopy. (**a**) Representative single-cell distance matrices and corresponding single-molecule 3D conformations of the HCT116 locus are shown for multiplexed FISH experiments [22] and for the corresponding LE and SBS models. (**b**) The similarity and structural variability of the conformational ensemble are quantified by genomic-distance-corrected pair correlations (*r*′) distributions derived from experimental and model single-molecule distance matrices. (**c**) The distributions of ellipsoid semi-axis ratios (*a*/*b* and *b*/*c*, schematically shown in the cartoon) derived from single-molecule conformations show that both models reproduce the main shape structural features observed experimentally. (**d**) The relative fractions of single-molecule compaction classes are also consistent between multiplexed FISH data and the LE and SBS model conformational ensembles. Hence, the models provide a quantitative description of DNA single-molecule conformational diversity. Adapted from [22,62].

**Figure 4:**
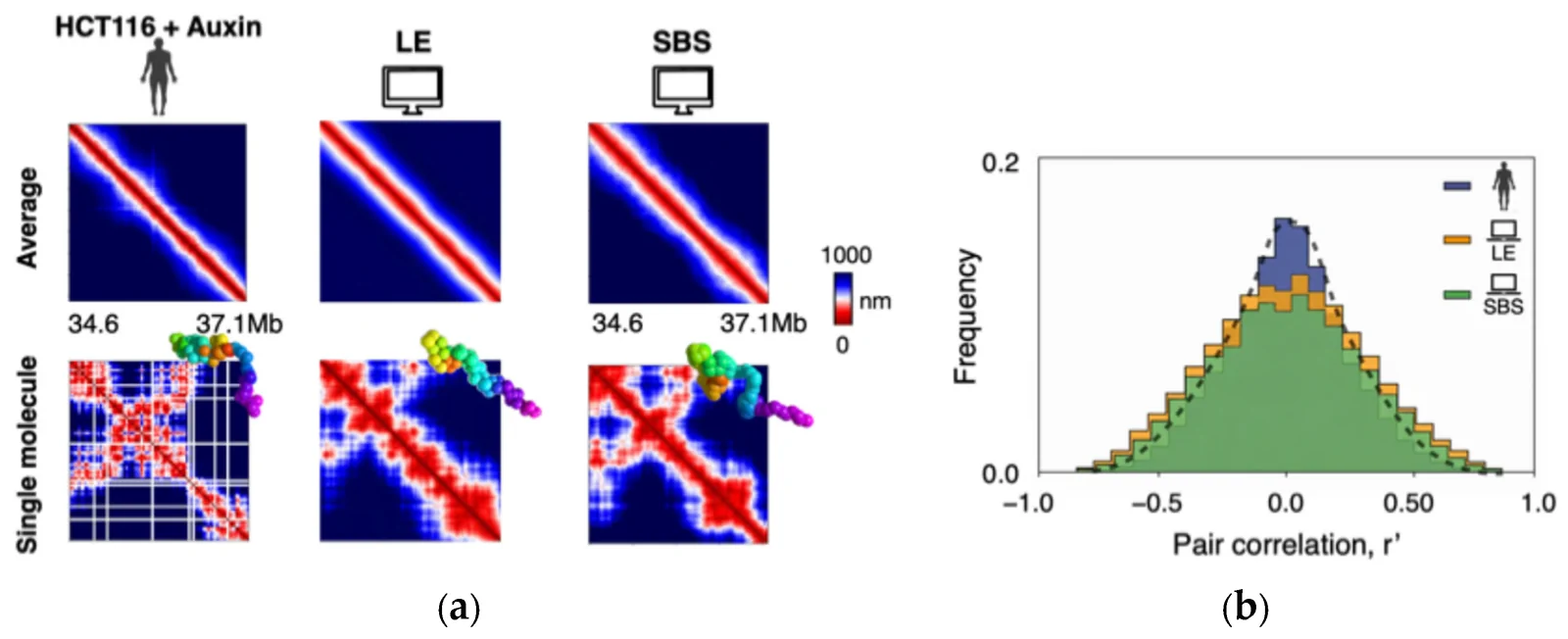
Cohesin-depleted single molecules have increased conformational diversity. (**a**) Population-averaged distance matrices and representative single-cell conformations of the HCT116 + Auxin locus are shown for multiplexed FISH experiments [22] and for the LE and SBS models. Despite the loss of pronounced population-averaged structural patterns, individual chromatin molecules retain heterogeneous non-trivial conformations that are reproduced by both models. (**b**) The structural heterogeneity of the conformational ensembles is quantified by the distributions of genomic-distance-corrected pair correlations, *r*′, between single-cell experimental structures and between model conformations. The broad distributions centered around values close to zero reflect the increased conformational diversity observed following cohesin depletion. Adapted from [61].

## Data Availability

Data are contained within the article.
